# Structural characterization of EGFR exon 19 deletion mutation using molecular dynamics simulation

**DOI:** 10.1371/journal.pone.0222814

**Published:** 2019-09-19

**Authors:** Mahlet Z. Tamirat, Marika Koivu, Klaus Elenius, Mark S. Johnson

**Affiliations:** 1 Structural Bioinformatics Laboratory, Biochemistry, Faculty of Science and Engineering, Åbo Akademi University, Turku, Finland; 2 Medicity Research Laboratories and Institute of Biomedicine, University of Turku, Turku, Finland; 3 Turku Bioscience Centre, University of Turku and Åbo Akademi University, Turku, Finland; 4 Turku Doctoral Programme of Molecular Medicine, University of Turku, Turku, Finland; 5 Department of Oncology and Radiotherapy, University of Turku and Turku University Hospital, Turku, Finland; Wake Forest University, UNITED STATES

## Abstract

Epidermal growth factor receptor (EGFR) is a tyrosine kinase receptor important in diverse biological processes including cell proliferation and survival. Upregulation of EGFR activity due to over-expression or mutation is widely implicated in cancer. Activating somatic mutations of the EGFR kinase are postulated to affect the conformation and/or stability of the protein, shifting the EGFR inactive-active state equilibrium towards the activated state. Here, we examined a common EGFR deletion mutation, Δ^746^ELREA^750^, which is frequently observed in non-small cell lung cancer patients. By using molecular dynamics simulation, we investigated the structural effects of the mutation that lead to the experimentally reported increases in kinase activity. Simulations of the active form wild-type and ΔELREA EGFRs revealed the deletion stabilizes the αC helix of the kinase domain, which is located adjacent to the deletion site, by rigidifying the flexible β3-αC loop that accommodates the ELREA sequence. Consequently, the αC helix is stabilized in the “αC-in” active conformation that would prolong the time of the activated state. Moreover, in the mutant kinase, a salt bridge between E762 and K745, which is key for EGFR activity, was also stabilized during the simulation. Additionally, the interaction between EGFR and ATP was favored by ΔELREA EGFR over wild-type EGFR, as reflected by the number of hydrogen bonds formed and the free energy of binding. Simulation of inactive EGFR suggested the deletion would promote a shift from the inactive conformation towards active EGFR, which is supported by the inward movement of the αC helix. The MDS results also align with the effects of tyrosine kinase inhibitors on ΔELREA and wild-type EGFR lung cancer cell lines, where more pronounced inhibition was observed against ΔELREA than for wild-type EGFR by inhibitors recognizing the active kinase conformation.

## Introduction

Epidermal growth factor receptor (EGFR) kinase is a tyrosine kinase involved in multiple cellular processes, such as cell proliferation, differentiation, migration and survival [1]. EGFR, also known as ErbB1/Her1, is a member of the ErbB family of receptor kinases, which also includes ErbB2, ErbB3 and ErbB4 (ErbBs). The ErbBs are fundamental to the development and growth of organisms, however, anomalies with their regulation and/or signaling activity often associates them with various cancers, making them key therapeutic targets [2, 3]. Already in the 1980’s it was observed that avian erythroblastosis retrovirus encoded chordate-species EGFR kinase domains that, untethered to a growth factor sensing ectodomain, was associated with development of cancers in the same chordate due to retrovirus infection [4, 5]. More recently, the extent of somatic mutations occurring in ErbBs from cancer patients has come to the forefront of research and patient treatment. For example, mutations in EGFR and amplification of ErbB2 predict sensitivity to EGFR and ErbB2 targeting cancer drugs, respectively [6, 7, 8]. Moreover, mutations observed in ErbB4 in non-small cell lung cancer patients experimentally lead to changes in ErbB2-ErbB4 heterodimer signaling promoting cell proliferation but not differentiation [9], and to increased phosphorylation likely because of stabilization of the ErbB4 active dimer state [10].

**Fig 1:**
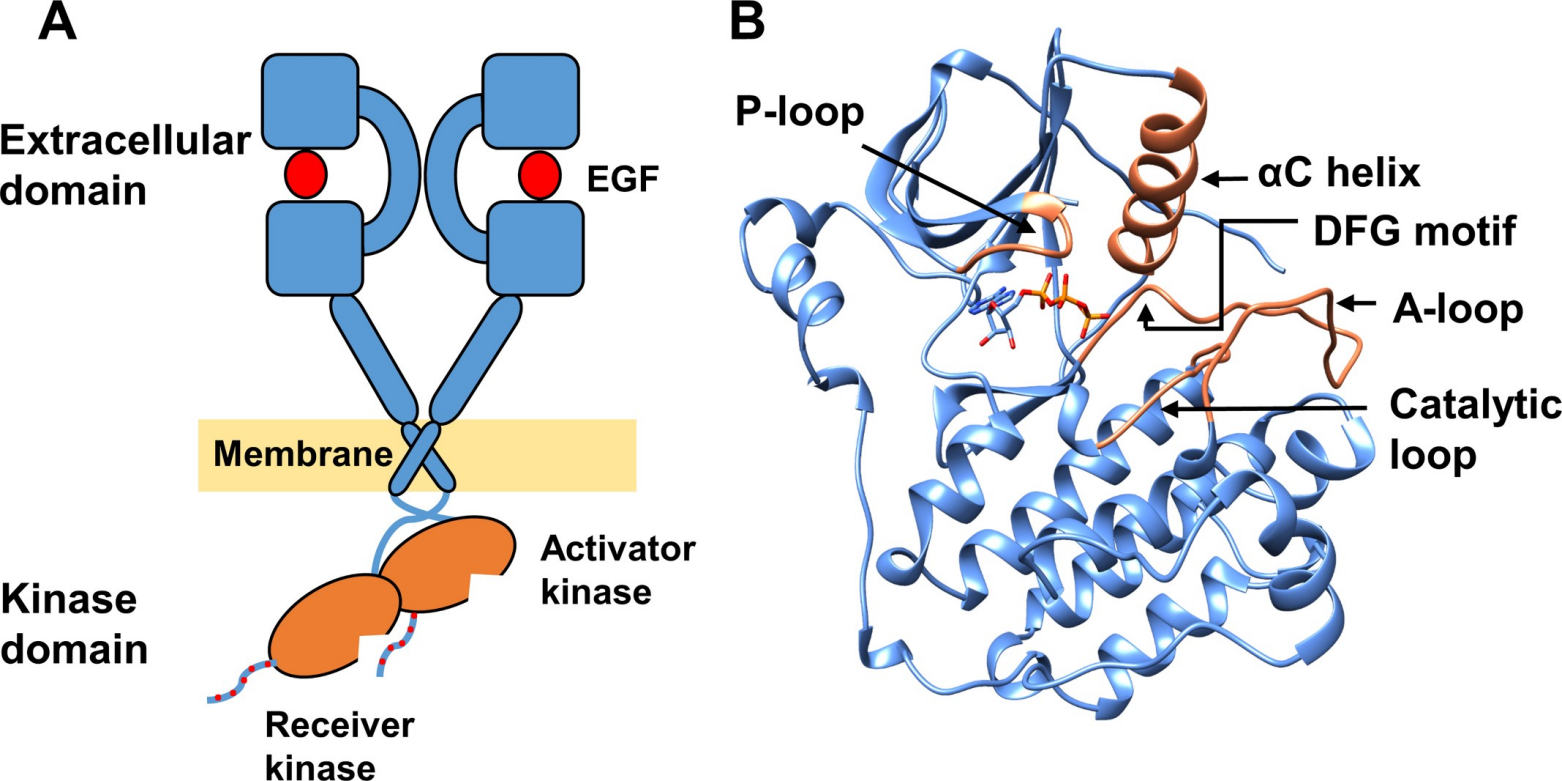
The EGFR structure. (A) EGFR dimer–growth factor (red circle) -bound extracellular domains, transmembrane domains and asymmetrically dimerized intracellular kinase domains (orange) with phosphotyrosines at the C-terminal tail marked in red dots. (B) The kinase domain of EGFR (PDB ID 2ITX). Missing loops have been built using other EGFR structures. Bound ANP in the crystal structure was replaced by ATP (sticks). Key structural features are shown in orange.

ErbBs are composed of an N-terminal extracellular growth factor binding domain, a helical transmembrane domain, a juxtamembrane sequence, an intracellular kinase domain that has an ATP binding / catalytic site, and a C-terminal tail that becomes phosphorylated during receptor activation (Fig 1A). ErbB activation is driven by growth factor binding to the ectodomain leading to dimer formation; ErbB2 itself does not bind growth factors but contributes to cell signaling by forming heterodimers with other EGFR family members that do bind the growth factor. The transition from monomer to either homodimer or heterodimer incurs a very large conformational change of the ectodomain. This in turn results in activation of the intracellular kinase domains through asymmetric dimer interactions, followed by autophosphorylation of tyrosine residues of the C-terminal tail, and triggering of multiple signal transduction pathways [1, 11–13].

The tyrosine kinase domain is a highly conserved bilobal structure consisting of the N-lobe and C-lobe, which are separated by the catalytic site where ATP binds (Fig 1B). The N-lobe is predominantly composed of β-sheets and constitutes key structural elements–such as the αC helix and P-loop–important for kinase activation and catalysis. The C-lobe, in contrast, is largely alpha helical and includes the functionally important activation and catalytic loops [14–16].

**Fig 2:**
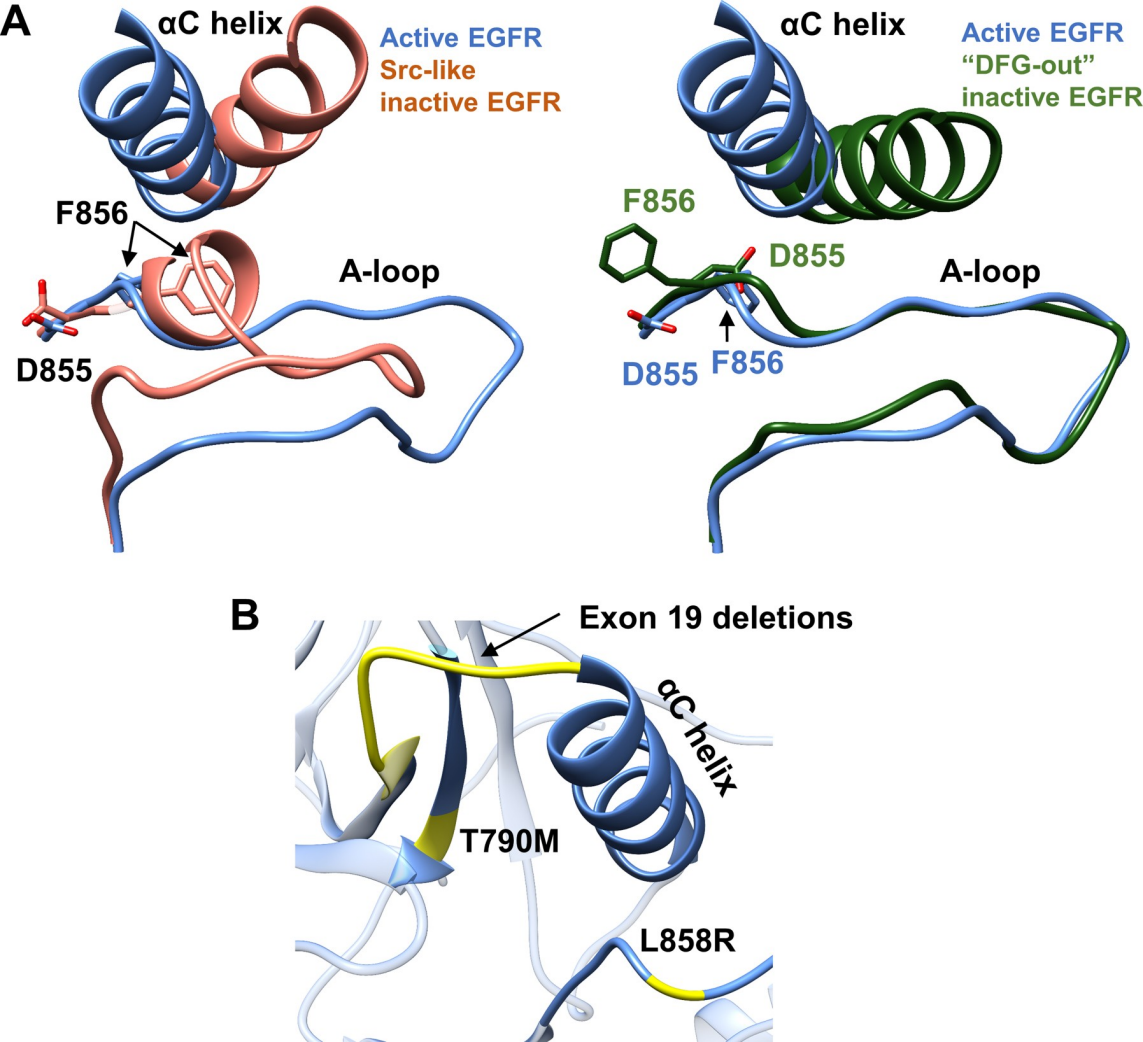
Active versus inactive structures of the EGFR kinase domain and common EGFR somatic mutations. (A) Superimposed structure of active EGFR (PDB ID 2GS2; blue) with (left) Src-like inactive EGFR (PDB ID 2GS7; orange); and (right) with DFG-out inactive EGFR (PDB ID 4I21; green). Aspartate and phenylalanine from the DFG motif are shown in sticks. (B) Main-chain location of the three common EGFR kinase somatic mutations (yellow) relative to the αC helix.

The EGFR kinase domain, like other kinases, is present in inactive and active conformations [17], the structures of which are well documented in the Protein Data Bank (PDB [18]). Monomeric ErbBs are inactive, but in the growth-factor induced dimer state the asymmetric interaction of the activator kinase domain and juxtamembrane segment B of the receiver kinase domain leads to the conformational changes required for phosphorylation [19]. The active and inactive conformations of kinase domain monomer structures mainly differ in the orientation of the αC helix and the activation loop (A-loop) (Fig 2A). In the active state, the αC helix is oriented towards the ATP binding pocket (“αC-in” conformation), forming an ion-pair interaction between a conserved glutamate and a lysine residue. Additionally, the A-loop that contains the aspartate-phenylalanine-glycine “DFG” motif attains an open and extended conformation, with the catalytic aspartate of the motif pointing towards the ATP binding site (“DFG-in” conformation). In contrast, two conformations of the inactive EGFR kinase are observed; the Src-like inactive state and the “DFG-out” state. The majority of the inactive EGFR structures in the PDB exist in the Src-like inactive conformation, where the αC helix is positioned away from the ATP binding site and assumes the “αC-out” position, breaking the conserved glutamate-lysine salt bridge. Moreover, this conformation exhibits a small hydrophobic helix at the N-terminus of the A-loop that packs against the αC helix, with the DFG motif in the DFG-in conformation. On the other hand, the DFG-out inactive conformation displays an αC helix that partly overlaps with the Src-like conformation and has an extended A-loop with a flipped DFG motif that positions the aspartate away from the binding pocket in the DFG-out conformation. These distinct conformational changes play a vital role in the regulation of kinase activity in the ErbBs [19–22].

Increased EGFR tyrosine kinase activity, due to over-expression and/or somatic mutation(s), has been linked to a variety of human cancers [23, 24]. In particular, activating mutations of the EGFR kinase domain, including single amino acid substitutions, insertions and deletions, are commonly observed in non-small cell lung cancer (NSCLC) patients [6, 7, 21, 25]. The most prevalent EGFR mutations in NSCLC are L858R and exon 19 deletions (Fig 2B), which respectively account for 41% and 44% of all EGFR mutations [17, 26]. Although NSCLC patients with these activating mutations respond to first generation tyrosine kinase inhibitors (TKIs), they often develop resistance due to a secondary T790M “gatekeeper” mutation [27, 28]. Unlike exon 19 deletions, 3D structures of EGFR with the L858R and T790M mutations have been determined [22, 29–31].

Exon 19 deletions have been reported to increase EGFR autophosphorylation, and promote cell survival by selectively activating AKT and STAT pathways [7, 32, 33]. Exon 19 deletions include a number of variants differing in the length of the deleted amino acid sequence, the most common subtype being Δ^746^ELREA^750^ [26, 34] (numbering from Uniprot ID P00533 and PDB ID 2ITX); and a majority of the subtypes share the deletion of the amino acids leucine-arginine-glutamic acid. These deletion mutations are located between the β3 strand and the αC helix within the β3-αC loop of the N-lobe of the kinase domain. The αC helix, positioned in the vicinity of the ATP binding / catalytic site and forming part of the asymmetric dimer interface, is fundamental for EGFR kinase activity. Hence, deletions within the β3-αC loop can be expected to exert their effects by altering local structural features on or about the αC helix, as well as by modulating tyrosine kinase activity and subsequent events.

Here, we have sought to understand the structural changes that take place as a result of the most prevalent exon 19 deletion in EGFR, ΔELREA, and the likely functional consequences due to changes in EGFR tyrosine kinase activity. To do so, the structures of the wild-type and mutant–active and inactive kinase domains–were assessed using molecular dynamics simulations (MDS) in order to probe the effects on domain conformation, local structure and consequences for biological activity.

## Materials and methods

### Structure preparation

X-ray structures of wild-type apo active (PDB code: 2GS2, 2.8 Å resolution; [19]), ATP-bound active (2ITX, 2.98 Å; [29]) and apo inactive (2GS7, 2.6 Å; [19]) served as a basis for the composite and model structures used in this study. The Src-like inactive structure is studied here as opposed to the DFG-out inactive conformation, on the basis that the majority of the experimentally resolved EGFR inactive structures are Src-like and include bound key TKIs unlike those of the DFG-out conformation. The bound ligand was removed from the apo inactive EGFR structure; for the ATP-bound active EGFR, the ligand ANP in 2ITX was replaced by ATP and a Mg^2+^ ion was added. Missing loops in all three structures were built using the loops from other EGFR structures (see S1 Table for details). Mutant ΔELREA EGFRs were then modeled for each of the above wild-type structures using the Modeller program [35] available in the Chimera visualization tool [36]. Altogether, this resulted in three wild-type and three ΔELREA EGFR structures, which were subsequently prepared for MDS using the protein preparation wizard in Maestro [37]: hydrogen atoms were added, optimal protonation states of ionizable side chains at pH 7.0 were determined using PROPKA [38], and the structures were energy minimized.

### Molecular dynamics simulation

Classical MDS was used to probe the dynamics of the six wild-type and ΔELREA EGFR structural models using the AMBER package (version 18) [39] and ff14SB force field [40]. Parameters for ATP [41] were used for the ATP-complexed systems. The proteins were solvated with explicit TIP3P water molecules [42] in an octahedral box, leaving 10 Å between protein surface atoms and edge of the box. The systems were neutralized by adding sodium counter ions. Additional Na^+^/Cl^-^ ions were added to the simulation box to achieve a 150 mM salt concentration. Periodic boundary conditions were employed and the particle-mesh Ewald algorithm [43] was used to treat electrostatic interactions with a distance cutoff of 9 Å. Prior to conducting the production simulation, 5000 cycles of steepest descent and conjugate gradient energy minimization were carried out. The minimization was initiated by introducing a 25 kcal mol^−1^ Å^−2^ restraint on solute atoms that was systematically reduced to 0 kcal mol^−1^ Å^−2^ over the total minimization step. The systems were then heated from 100 K to 300 K during 100 ps with a 10 kcal mol^−1^ Å^−2^ restraint on solute atoms. Subsequently, a 900 ps equilibration at constant pressure was employed while reducing the restraint gradually to 0.1 kcal mol^−1^ Å^−2^. The equilibration protocol was concluded with an unrestrained 5 ns simulation. Finally, the production simulation was carried out for 100 ns at constant temperature (300 K) and pressure (1 bar) that was maintained using the Berendsen algorithm [44] with 5 ps coupling constant. In order to sample more conformational space, the simulations were performed in triplicate using different initial velocities, assigned by a pseudo-random number generator. Coordinates were saved every 10 ps and the resulting trajectories were analyzed further using the programs CPPTRAJ [45] and VMD [46]. CPPTRAJ was used to compute Cα-atom root-mean-squared fluctuations (RMSF) and to examine hydrogen bonds. A hydrogen bond was defined as a donor-acceptor distance of less than or equal to 3.5 Å and a bond angle of greater than or equal to 135°. The root-mean-squared deviation (RMSD) was calculated over Cα atoms using Chimera. VMD was critical for visualizing trajectories and monitoring distances between residues.

### Principal component analysis

Principal component analysis (PCA) is a multivariate statistical method that was used in this study to reveal any dominant patterns of motion recorded during MDS. Initially, the recorded MD trajectory frames were superimposed on the average structure to remove global translational and rotational motions. Subsequently, a coordinate covariance matrix was generated for backbone atoms using the 3D positional coordinates from the trajectory frames. Diagonalizing this matrix generates eigenvectors and corresponding eigenvalues that respectively describe the direction and magnitude of motion. In this study, PCA was carried out for backbone atoms of the αC-helix and β3-αC loop in both wild-type and ΔELREA active EGFRs using the program CPPTRAJ. The normal mode data generated for the top three principal components (PC) were analyzed using the Normal Mode Wizard (NMWiz) [47] included in VMD.

### Free energy calculations

To assess the relative binding free energy, ΔG_bind,_ of ATP with the wild-type and ΔELREA EGFRs, the molecular mechanics generalized Born surface area (MM-GBSA) module [48] available in the AMBER package was applied to the resultant trajectories from the last 50 ns of the simulation. The MM-GBSA method can be summarized as follows:

$$\Delta G_{bind}=G_{complex}‐(G_{protein}+G_{ligand})$$

Where G_complex_ is the free energy of the protein-ligand complex, G_protein_ is the free energy of the protein and G_ligand_ is the free energy of the ligand.

### Drug response data for the EGFR ΔELREA mutant

Drug response data from three publicly available databases, The Cancer Cell Line Encyclopedia (CCLE) [49], The Genomics of Drug Sensitivity in Cancer (GDSC) [50], and The Cancer Therapeutics Response Portal (CTRP; second version) [51] were downloaded from the websites https://portals.broadinstitute.org/ccle, http://www.cancerrxgene.org/, and https://ocg.cancer.gov/programs/ctd2/data-portal. RStudio version 3.5.1 [52] was used to sort through the data and to collect drug response data for lung cancer cell lines that contain the EGFR ΔELREA mutation and for lung cancer cell lines that are wild-type for all four ErbB receptors, EGFR, ErbB2, ErbB3, and ErbB4 (“EGFR wild-type”). Mutations in other ErbBs, in addition to EGFR, may affect the sensitivity to EGFR/ErbB inhibitors. Thus, only ErbB wild-type cell lines were included in the control group to reduce the possibility of the control response curve shifting due to known and unknown ErbB mutations other than the EGFR ΔELREA mutation. To further control the effect of including or excluding the ErbB2, ErbB3 and ErbB4 mutant cell lines from the control group, the mean area-under-the-curve (AUC) values were calculated for both the cell lines wild-type for EGFR irrespective of the mutation status of ErbB2, ErbB3 and ErbB4, and for cell lines wild-type for all four ErbBs. The values were very similar to each other (data not shown). The R code executed to process the data, including the libraries used, is provided as a supplementary document (S7 File). One hundred percent cell survival was assigned to a concentration of 0 μM and drug response values were transformed into percentages. The analyses were carried out for the ErbB tyrosine kinase inhibitors erlotinib, gefitinib, afatinib, and lapatinib.

## Results

Kinases are dynamic proteins that exist in an equilibrium between active and inactive states, and access to the active catalytic state is highly regulated and of restricted duration in order to prevent signaling effects that lead to abnormal biological effects. Whereas kinases exhibit similar active state conformations, individual kinases can adopt different inactive conformations [15, 16].

**Fig 3:**
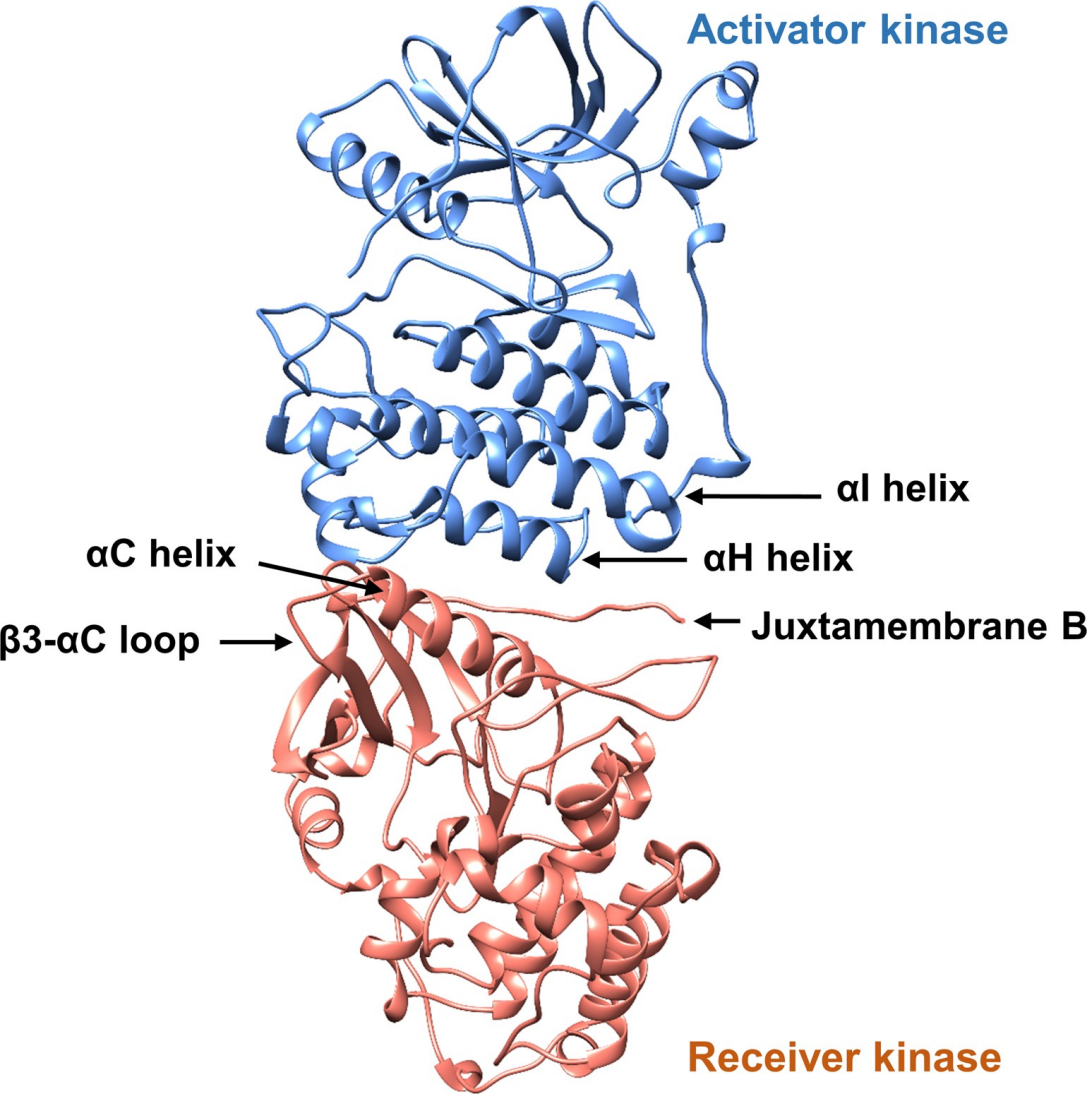
The EGFR asymmetric dimer. Interaction between the activator (blue) and receiver (orange) kinase domains in the EGFR asymmetric dimer; key structural elements at the dimer interface are highlighted, as well as the β3-αC loop.

Like other kinases of the receptor tyrosine kinase family, EGFR forms a dimer on binding the growth factor ligand to the ectodomain, serving to bring the cytoplasmic kinase domains together to form an asymmetric complex and leading to kinase activation. The interface of the kinase asymmetric dimer involves interactions of the αC helix with the juxtamembrane B peptide from the receiver kinase domain and helices αH and αI of the activator kinase domain (Fig 3). The β3-αC loop from the receiver kinase domain is not itself in contact with the activator kinase domain, but this loop directly precedes the αC helix that is in direct contact.

Here, we investigated a commonly observed activating EGFR kinase deletion, ΔELREA, in order to evaluate any structural changes resulting from the deletion reported to increase kinase activity [53, 54]. All-atom MDS was employed on apo forms of wild-type and ΔELREA EGFRs, both in the active and inactive conformations. Additionally, simulation of wild-type and ΔELREA EGFR-ATP complexes was carried out in order to determine the dynamic effect of the mutation on ATP binding. The simulations were performed in triplicate with different initial velocities and consistent observations were recorded, although simulation 2 of both ATP-bound wild-type and mutant EGFR showed a wider range of motions and larger estimated free energy of binding. Here we have described the results from simulation 1.

### Active EGFR kinase: Dynamics of wild-type and ΔELREA EGFR

**Fig 4:**
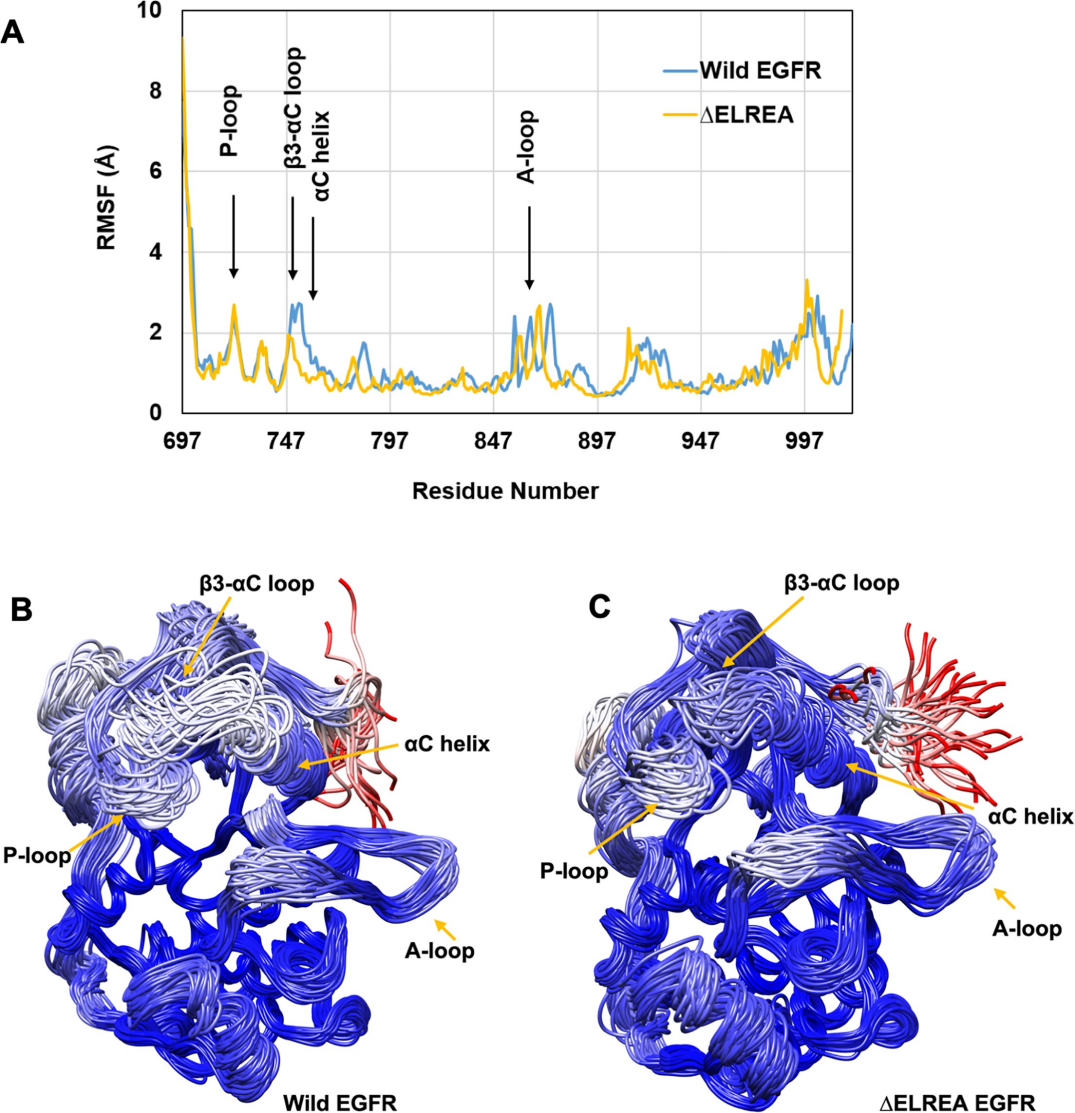
Cα-atom fluctuations and conformational ensemble of active wild-type and ΔELREA EGFRs. (A) RMSF calculated over Cα atoms for conformations sampled from MDS, showing large fluctuations for unstructured regions in both EGFRs; wild-type (Wild EGFR) and mutant (ΔELREA) EGFR are offset by 5 residues starting from residue 746. Superimposed conformations of wild-type (B) and ΔELREA (C). Chain traces are colored based on RMSDs for individual Cα atoms within the ensemble according to the program Chimera: The gradient of coloring varies from blue with RMSD = 0.45 Å (minimum observed value), to white at 5.6 Å and to red = 11 Å (maximum observed value).

In order to assess the overall dynamics of wild-type and ΔELREA EGFRs during the simulations, the RMSF (Cα atoms) was computed for each saved trajectory: it is evident that the loop regions fluctuate more than the secondary-structured regions in both EGFRs (Fig 4A). These loops include the N-terminal juxtamembrane segment, P-loop, A-loop and the C-terminal end of the proteins (note that due to the 5-residue deletion in ΔELREA, the plots for wild-type and mutant EGFR (Fig 4A) are correspondingly offset from each other beginning from residue 746). A significant difference in fluctuation between wild-type and mutant EGFRs occurs at the β3-αC loop–where the deletion mutation is located–and along the adjacent αC helix. These regions fluctuate more in wild-type EGFR in comparison to ΔELREA. With the DFG motif there was no measurable change occurring as a result of the mutation, likely because only the side chain of F856 is in contact with the C-terminal end of the αC helix, which is itself positionally stabilized.

To better visualize the dynamic movements taking place in wild-type and ΔELREA EGFRs, conformations sampled from the simulations over a given simulation-time interval were retrieved and superimposed on the median structure. The backbone traces are color coded according to the RMSD values over Cα atoms for the conformational ensembles versus the median structure: blue indicates positionally stabile regions, whereas white and red represent increased mobility. Both the wild-type (Fig 4B) and ΔELREA (Fig 4C) EGFRs share similar stability profiles over a majority of each structure, with the exception of the β3-αC loop and the αC helix. These regions appear to be less mobile in the mutant EGFR (average RMSD 1.91 Å) in comparison to wild-type EGFR (average RMSD 2.93 Å), a clear indication of the impact of the ΔELREA deletion.

### Active EGFR kinase: ΔELREA constrains the αC helix

**Fig 5:**
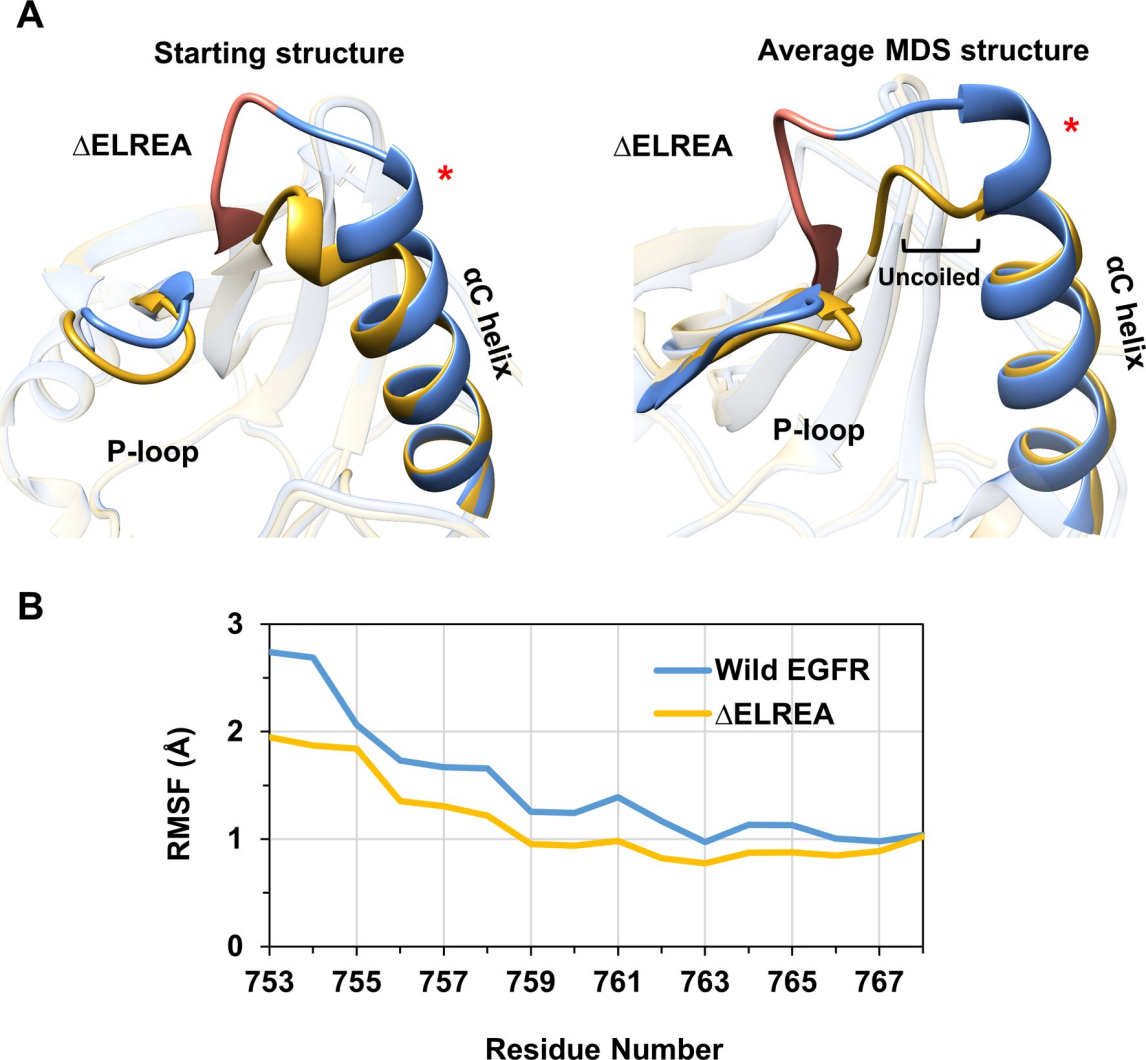
Wild-type vs ΔELREA active EGFR structures and RMSF of the αC helix. (A) Superimposed active EGFR starting structure (left) and average MDS structure (right): wild-type (blue) and ΔELREA (gold), highlighting the αC helix, P-loop and the deleted ΔELREA section (orange). The N-terminal end of the αC helix is marked with a red asterisk in all relevant figures as a point of reference. (B) Cα**-**atom RMSF over residues of the αC helix for the wild-type and mutant EGFR structures with respect to the average structure.

In the initial structural model of the active form ΔELREA EGFR (Fig 5A, left), as a result of the deletion, the N-terminal portion of the αC helix (residues 753–755) deforms and relocates to the position formerly occupied by the β3-αC loop. Similar features have been observed in X-ray structures of human B-RAF serine/threonine kinase with a β3-αC loop deletion mutation, where this loop was highlighted as a critical feature–“a rheostat”–modulating kinase activity [55].

During the simulation of the active form ΔELREA EGFR, the N-terminal part of the αC helix, nearest the site of the deletion, is uncoiled, as depicted by the average structure from the simulation of ΔELREA EGFR (Fig 5A, right). The structural changes arising from ΔELREA also places the αC helix in close proximity to the phosphate binding P-loop. This loop is involved in coordinating the nucleotide substrate, covering the ATP binding site where the α and β phosphates reside. During the simulation of wild-type active EGFR, Phe723 of the P-loop is on average 11.6 Å (95% confidence interval (CI) ± 0.04) from Ile759 of the αC helix (measured between Cα atoms). This distance is 1.5 Å shorter in the mutant, averaging 10.1 Å (95% CI ± 0.04). The P-loop also packs closely with the uncoiled N-terminus of the αC helix, which in turn reduces the flexibility of the uncoiled helix.

The Cα atoms of the αC helix (residues 753–768) fluctuate less in the mutant (average RMSF of 1.1 ± 0.4 Å) than in wild-type EGFR (average RMSF of 1.5 ± 0.57 Å) in comparison to their respective average structures (Fig 5B). This result is consistent with the shortened linker loop of ΔELREA EGFR between strand β3 and the αC helix that constrains and limits the movement of the helix with respect to the strand. It is noteworthy that the β3-αC loop is often missing in X-ray structures of the EGFR kinase domain, a testament to the flexibility of the wild-type loop. Deletion of a section of this loop would then help rigidify the loop and stabilize the succeeding αC helix in the “αC-in” conformation, which would in turn shift the equilibrium in favor of the enzymatically active state EGFR and likely prolonging the lifetime of that activated state, too.

**Fig 6:**
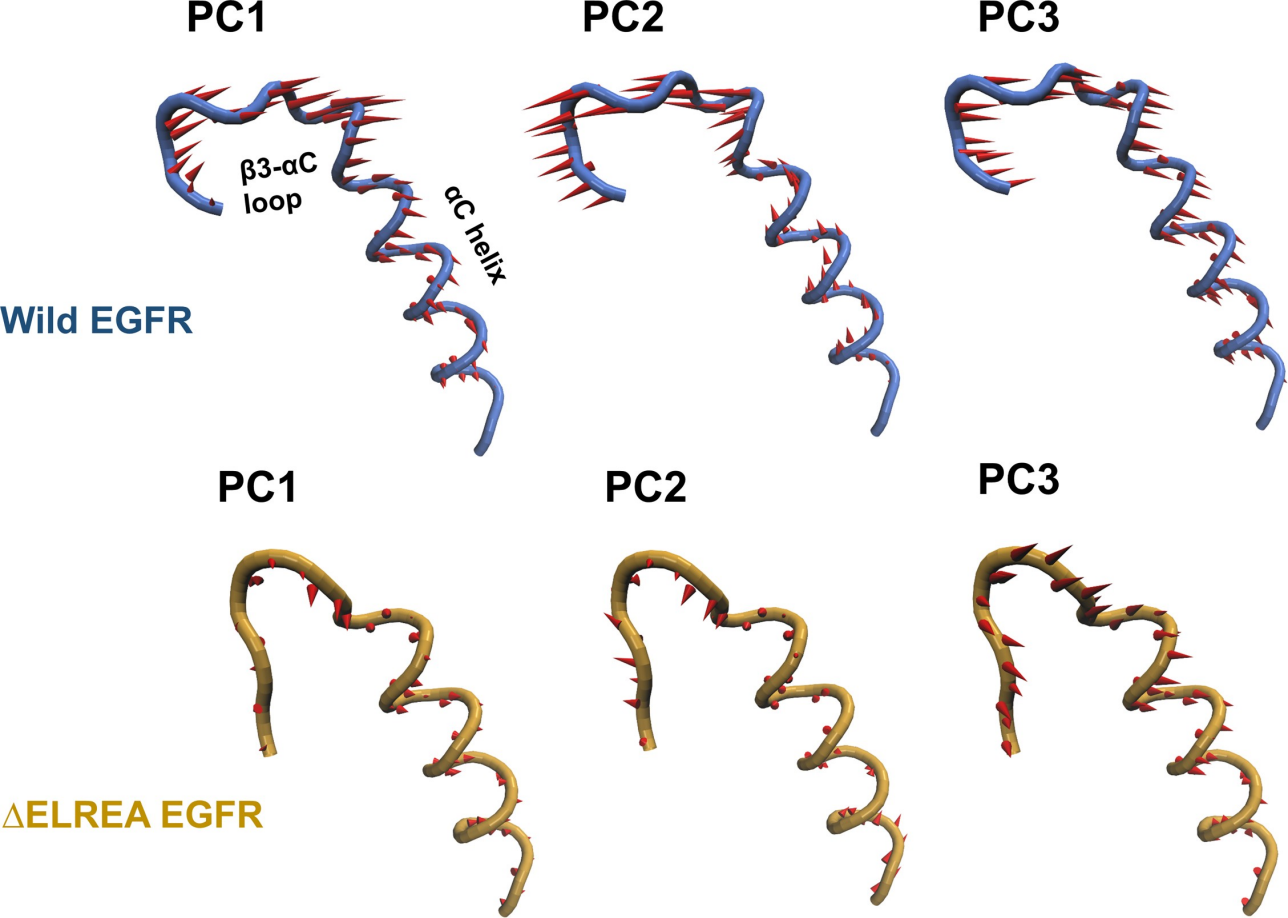
Principal motions of wild-type and ΔELREA EGFR. The top three PCs for the backbone atoms of the αC helix and β3-αC loop of wild-type (blue) and ΔELREA (gold) EGFR are shown with a “porcupine” plot; red arrows represent the magnitude and direction of motion.

The principal motions revealed in the PCA calculations for the backbone atoms of the β3-αC loop and αC helix (Fig 6) also indicate higher mobility over these structural units in wild-type EGFR relative to the ΔELREA mutant. The top three PCs for β3-αC loop and αC helix of wild-type and ΔELREA EGFRs are represented as “porcupine” plots, where the arrows represent the direction and magnitude of motions over the backbone atoms. In ΔELREA EGFR, both the αC helix and the remaining portion of the β3-αC loop appear to be stabile over all three PCs as indicated by the size of the arrows, in contrast to wild-type EGFR, exhibiting relatively large motions for both the αC helix and β3-αC loop. Furthermore, the αC helix motions of wild-type EGFR are directed upwards and outwards, which may drive the conformational transition from the “αC-in” to the “αC-out” state.

Although the ΔELREA deletion is not in direct contact with the asymmetric dimer interface (Fig 3), the deletion could also have an indirect effect on the dimer interaction through the stabilization exerted on the αC helix. The αC helix of the receiver kinase along with the juxtamembrane B segment is an integral part of the dimer interface, interacting with the αH and αI helices of the activator kinase. Therefore, it can be hypothesized that the deletion within the β3-αC loop may also stabilize the interactions of the EGFR asymmetric dimer interface, and hence lead to a prolonged activation.

### Active EGFR kinase: ΔELREA and the E762^…^K745 salt bridge

**Fig 7:**
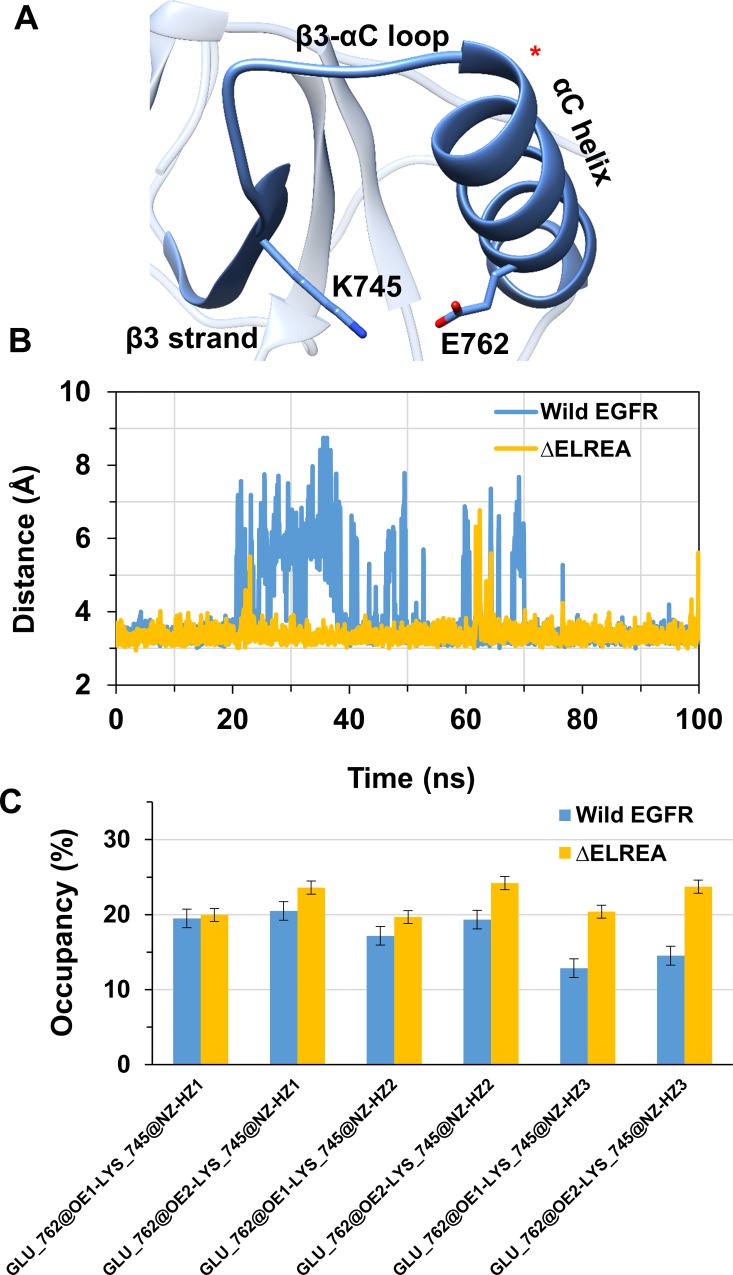
EGFR E762^…^K745 salt bridge behaviour during the MDS. (A) Key salt bridge formed between K745 and E762 in the active EGFR kinase (shown in sticks). (B) Distance between K745 and E762 during the 100 ns simulation. Wild-type active EGFR (blue) displays longer distances and more fluctuations in the distances between C*δ* of Glu762 and Nζ of Lys745 of the ion pair as compared to ΔELREA EGFR (gold). The C*δ*⋯Nζ distances of the salt bridge are provided in S1A Fig. C) Percentage occupancy of hydrogen bonds between terminal hydrogen atoms HZ1-3 of K745 and carbonyl oxygen atoms Oε1 and Oε2 of E762 in the wild-type and ΔELREA EGFRs.

A salt bridge conserved among kinases, forming between a glutamate from the αC helix and a lysine from the β3 strand, is fundamental for tyrosine kinase activity. The ionic interaction helps to optimally orient and stabilize the lysine, which in turn interacts with the α- and β- phosphates of ATP, placing them properly for catalysis [14, 15] (Fig 7A). The E762^…^K745 salt bridge is present when the kinase is in the αC-in active conformation; conversely, the ionic interaction is broken and phosphate transfer is disrupted when the helix is oriented outwards as in the inactive conformation [16, 20]. To assess the dynamics of this interaction in the EGFR active state, we monitored the distance between the side-chain atoms C*δ* of Glu762 and Nζ of Lys745 in the wild-type and ΔELREA trajectories (see S1A Fig).

With active ΔELREA EGFR, the 4.2 ± 0.2 Å (95% CI ± 0.01) average distance between the side-chain atoms of the salt bridge is clearly more consistent and less variable than the 4.7 ± 1.0 Å (95% CI ± 0.04) observed in the simulation of wild-type active EGFR (Fig 7B). This reflects the mutual stabilization of both the αC helix and β3 strand relative to each other due to the reduction of the intervening loop and consequential support for the formation and maintenance of the salt bridge that links both secondary structures to each other. The simulation of wild-type active EGFR results in longer E762 and K745 distances in multiple frames of the trajectory, implying frequent disruption of this interaction. The percentage occupancy for hydrogen bonds formed by the side-chain polar atoms of E762 and K745 also support this view (Fig 7C): the Glu762 –Lys745 hydrogen bond is observed in more frames of the mutant trajectory than for wild-type EGFR. These findings suggest that the stability of the αC helix imparted by ΔELREA mutation also enhances the stability of the E762^…^K745 salt bridge, which is key to EGFR kinase activity.

### Active EGFR kinase: ΔELREA and ATP binding

**Fig 8:**
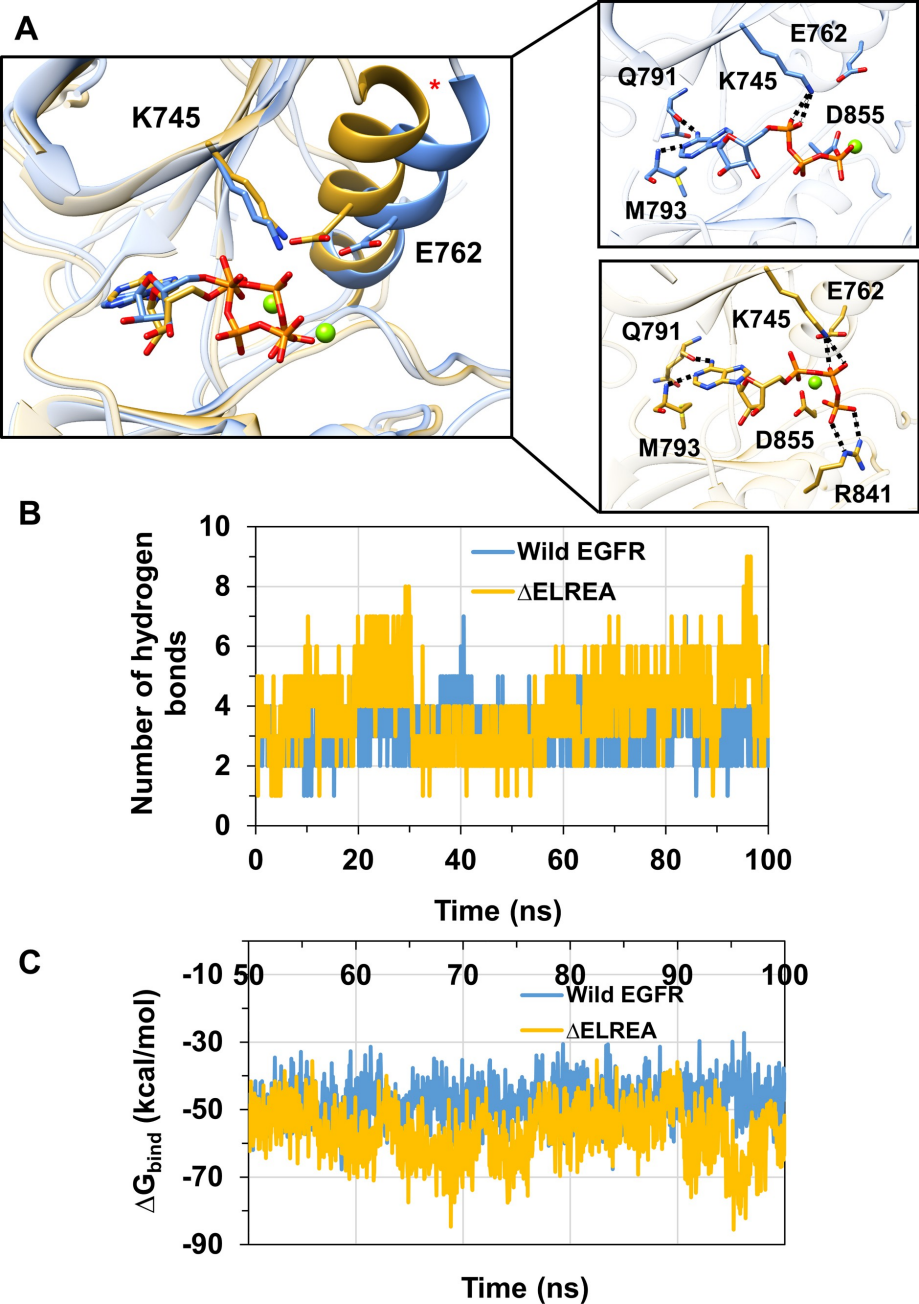
ATP binding to wild-type and ΔELREA EGFR. Average structures of the (A) wild-type (blue) and ΔELREA (gold) EGFR-ATP complexes from the MDS. The individual αC helices fluctuate more in wild-type EGFR than ΔELREA EGFR (S2 Fig). The orientation of the triphosphate group of ATP and location of Mg^2+^ alternate relative to the side-chain location of D855 in the simulations. ATP is shown in the binding pocket with a magnesium ion (green) along with key residues interacting with Mg^2+^–ATP or involved in catalysis; and hydrogen bond interactions observed frequently (dotted line). (B) The number of hydrogen bonds formed between ATP and the two EGFRs during the 100 ns simulation. (C) Binding free energy of ATP to wild-type and ΔELREA EGFR during the 100 ns simulation. The number of hydrogen bonds observed between ATP and both wild-type and ΔELREA EGFR, as well as the recorded binding free energy, is shown in S1B and S1C Fig.

Protein kinases catalyze the phosphorylation of proteins fueled by Mg^2+^–ATP. The cation coordinates with phosphate groups of ATP, helping to neutralize the negative charge, and optimally orienting the nucleotide for γ-phosphate transfer [56]. The adenosine ring of ATP binds in a hydrophobic pocket near the hinge region of EGFR, and the phosphate groups extend towards the N-terminal part of the A-loop (Fig 8A). During the simulation of the Mg^2+^–ATP complexes of active ΔELREA and wild-type EGFRs, a substantial difference is observed in the conformation of the αC helix, which moves away from the binding pocket in the case of wild-type EGFR (Fig 8A). In contrast, the αC helix of the ΔELREA deletion mutant maintains its initial position near the active site, owing to the physical restraint introduced by the deletion. The orientation of the triphosphate moiety of ATP and the location of Mg^2+^ varies within all of the simulations of both mutant and wild-type EGFR (Fig 8A). Thus, these differences do not correlate with the ΔELREA mutation, but instead follow the two observed orientations of the D855 side-chain from the DFG motif that coordinates Mg^2+^.

The most stable interactions in both EGFR-ATP complexes occur between backbone atoms of Gln791 and Met793 and the adenosine ring of ATP (Fig 8A). These interactions exist in more than 90% of the snapshots obtained from the simulation. A key interaction which is differentially observed in the two EGFR-ATP forms takes place between Lys745 of the conserved salt bridge and phosphate groups of ATP. This interaction is more preserved in ΔELREA EGFR, likely a result of a more stable αC helix (average Cα atom RMSF of 0.5 Å), which in turn stabilizes the salt bridge between Glu762 and Lys745, therefore optimally positioning Lys745 to make the interaction with ATP. In contrast, with wild-type EGFR the full-length β3-αC loop adds local flexibility and imposes fewer restraints, resulting in a less conformationally stabile αC helix (average Cα atom RMSF of 1.4 Å) and salt bridge between Glu762 and Lys745. As a result, Lys745 is not as strictly available as in the mutant EGFR to interact with the phosphate groups of the nucleotide. ΔELREA EGFR also benefits from a well-maintained hydrogen bond between ATP and Arg841, which is less frequently observed in wild-type EGFR (see S2 Table for details). The number of hydrogen bonds within a distance of 3.5 Å formed between ATP and the two EGFRs during the simulation can provide a general view of the magnitude of interactions taking place: an average of 4.0 (95% CI ± 0.03) hydrogen bonds were observed for the mutant and 3.2 (95% CI ± 0.04) for wild-type EGFR (Fig 8B).

To estimate the relative binding affinity of ATP towards the wild-type and ΔELREA EGFR kinases in the active conformation, the free energy of binding of the nucleotide was computed using the MM-GBSA method. The result (Fig 8C) shows that ΔELREA EGFR exhibits a lower binding free energy, average ΔG_bind_ of -57 kcal/mol (95% CI ± 0.43), as compared to the wild-type, which has an average ΔG_bind_ of -48 kcal/mol (95% CI ± 0.33). This difference in free energy of binding is consistent with the larger number of favorable hydrogen bonds observed in the ΔELREA EGFR-ATP complex in comparison to the wild-type. In summary, these findings support the notion that active ΔELREA EGFR would have a higher binding affinity for ATP than the active wild-type EGFR, which in comparison to the active wild-type EGFR may be a contributing factor to the experimentally observed increased activity of the deletion mutant [53, 54].

### Effect of ΔELREA on the inactive EGFR kinase

The dynamic switching between inactive and active conformations of kinases is a fundamental process contributing to the regulation of their activity [15]. In ErbBs, it is not only the activation triggered by dimerization on binding growth factor, but also changes from the active to inactive state that can dampen down ErbB phosphorylation and signaling. Furthermore, the relative stablization of features of the inactive versus active conformations can shift the equilibrium towards the more stabile state, increasing its lifetime. Hence, it is of great importance to assess the possible structural effects of ΔELREA not only on the active, but also on the inactive EGFR kinase.

**Fig 9:**
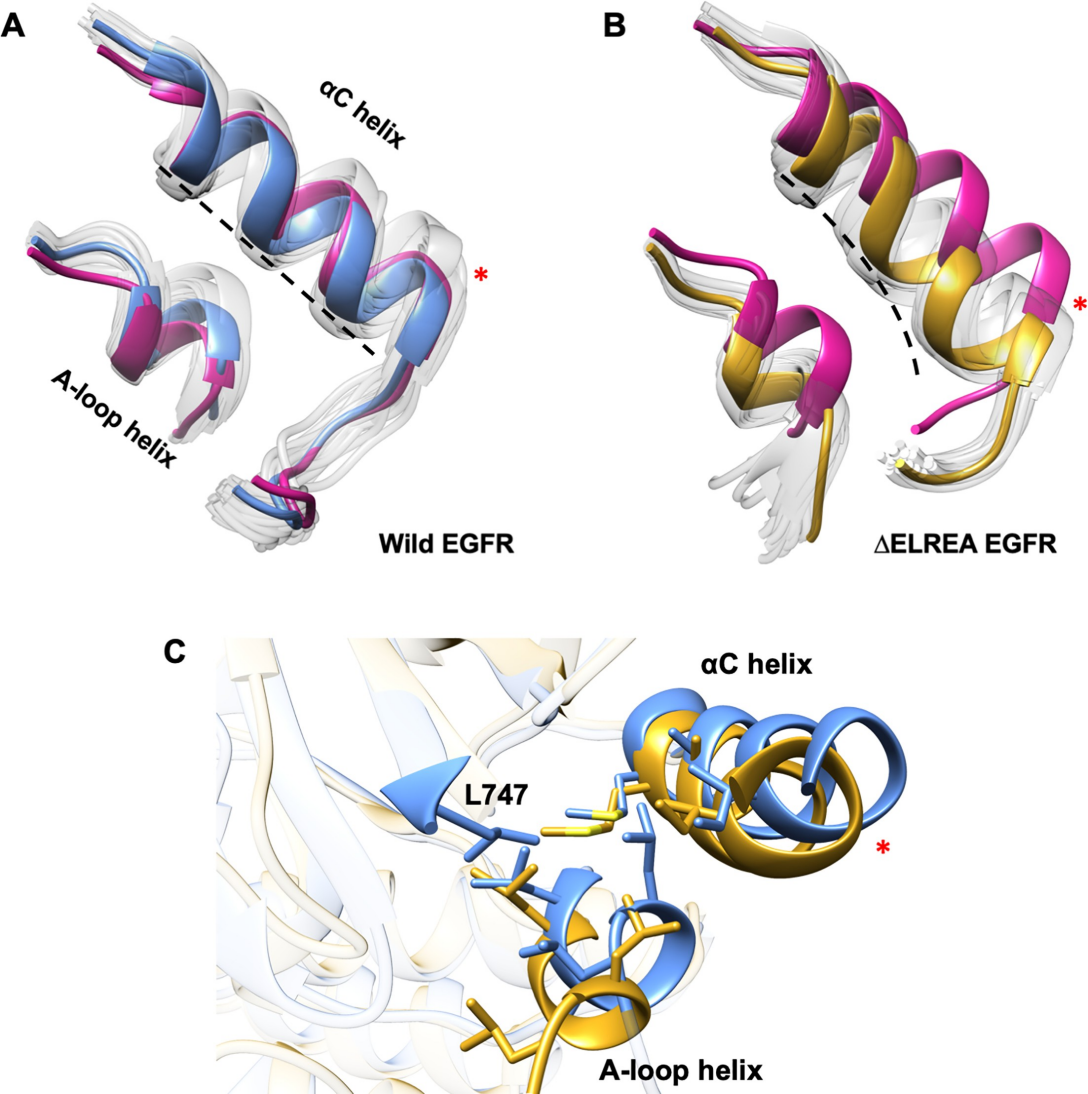
Inactive form of wild-type versus ΔELREA EGFR during MDS. Superimposed conformations of the A-loop and αC helices of wild-type (A) and ΔELREA (B) inactive EGFRs sampled during MDS. The median structure in the wild-type and mutant EGFRs is highlighted in blue and gold, respectively, initial unminimized structures in pink, conformational variations among sampled structures in transparent white (view inspired by [57]). Wild-type EGFR maintains the initial positions of the A-loop and αC helices, which are displaced in the mutant EGFR. The αC helix of the wild-type EGFR remains linear whereas the mutant is curved (dotted line). (C) Amino acids contributing to the hydrophobic network between the αC helix and the A-loop helix are shown as sticks. L747 from the β3-αC loop, which is deleted in the ΔELREA mutant, is labeled.

In contrast to active EGFR structures, the inactive structures present in the PDB exhibit an αC helix with an uncoiled N-terminus. Hence, both the wild-type and mutant inactive EGFR structures used for the MDS are uncoiled at residues 753–755, which are now part of the β3-αC loop. The only difference between the wild-type and ΔELREA inactive EGFR initial models is therefore the length of the β3-αC loop, which is shorter for the latter as a result the deletion. To assess the effect of the mutation on the inactive structures during the simulation, the initial unminimized inactive structures and sampled conformations from both the wild-type EGFR and ΔELREA inactive EGFR were superimposed on their respective median structures (solid colors, Fig 9A and 9B). With ΔELREA a striking effect of the mutation is observed: the αC helix moves “inwards” towards the ATP cleft, a movement required to attain the active state conformation. In contrast, wild-type EGFR largely maintains the initial position of the αC helix. Moreover, the conformational ensemble (transparent chain-traces) shows a small “outward” motion for the wild-type αC helix (also for the A-loop helix) as compared to the initial unminimized structure. The αC helix of the mutant also becomes bent during the simulation, which is not observed for wild-type inactive EGFR where the central axis of the helix remains linear. The bent conformation in ΔELREA results from the mutation-induced inward movement of the αC helix, which is then blocked by the small hydrophobic A-loop helix that packs against the αC helix and hinders a full inward movement (Fig 9B). Additionally, due to the partial inward movement of the ΔELREA αC helix, the A-loop helix is “pressed down” from its initial position, making the distance between the two helices wider as compared to the wild-type inactive EGFR that maintains the initial positions of the two helices (Fig 9A). This notion is reflected in the distance between the Cα atoms of Ile759 of the αC helix and Leu862 of the A-loop helix during MDS, averaging 5.8 Å (95% CI ± 0.01) for wild-type and 7.4 Å (95% CI ± 0.02) for mutant EGFR. Furthermore, compared to the wild-type inactive EGFR, the mutant displays a “loose” hydrophobic network between the two helices owing to the deletion of Leu747 of the β3-αC loop (ΔELREA), which normally contributes to the hydrophobic interaction in the wild-type EGFR (Fig 9C). Consequently, disruption of the obstructing hydrophobic cluster would be feasible in the mutant inactive kinase, which would predispose it to transition towards the active conformation.

It is noteworthy that observing large conformational changes in macromolecules requires longer simulations, in the range of micro and milliseconds that are computationally demanding. The same length of simulation time is needed to observe the transition among the conformational states of the EGFR tyrosine kinase [58]. Given our simulations were in nanosecond timescale, we do not expect to observe ΔELREA driven conformational changes from the inactive to the active EGFR state. Nonetheless, from the 100 ns simulation it can be stipulated that ΔELREA mutation favors an inward movement of the inactive EGFR αC helix and the mutation interferes with the obstructing hydrophobic cluster, features that support a shift from the inactive conformation towards that seen in active EGFR.

## Discussion

EGFR is a large, complex receptor protein involved in various signaling pathways that regulate cell proliferation, migration and apoptosis [1]. The activity of EGFR and its family members are normally highly regulated and complex, since it is apparent that many regions of the structure can influence EGFR signaling and its active lifetime. Indeed, mutations in the extracellular, transmembrane and intracellular domains have been shown to enhance tyrosine kinase phosphorylation as well as cause changes in signaling by altering the active-inactive dynamics of the protein [2, 17, 26, 59]; over-expression, unregulated retroviral tyrosine kinase domains, and somatic mutations are all associated with cancers [23, 24].

In this study, we examined the ΔELREA deletion mutation in human EGFR that is commonly observed in NSCLC patients. Indeed, ΔELREA, located at the β3-αC loop of the kinase domain, is reported to result in increased kinase activity [53, 54]; and of variable-length deletions introduced into the β3-αC loop of EGFR, a five-residue deletion led to maximal activity [55]. Using MDS, we investigated the implications of the mutation on the active and inactive EGFR kinase structures, and the effect of ΔELREA on ATP binding. In the active kinase structure, the mutation was shown to stabilize the αC helix in the “αC-in” conformation by constraining its movement due to deletion of a section of the flexible β3-αC loop. This would help maintain the salt bridge between Glu762 and Lys745 that is crucial for kinase activity. The deletion also results in a higher affinity interaction between ATP and EGFR, which is supported by a lower binding free energy value for the mutant-ATP complex in comparison to wild-type EGFR. Moreover, in the inactive kinase structure, ΔELREA results in an inward movement of the αC helix that also disrupts a hydrophobic barrier between the active site and the αC helix, which would advocate a shift from an inactive conformation to the active conformation of EGFR. Each of these observations from MDS is consistent with a kinase domain with increased stability and lifetime in the active catalytic state.

**Fig 10:**
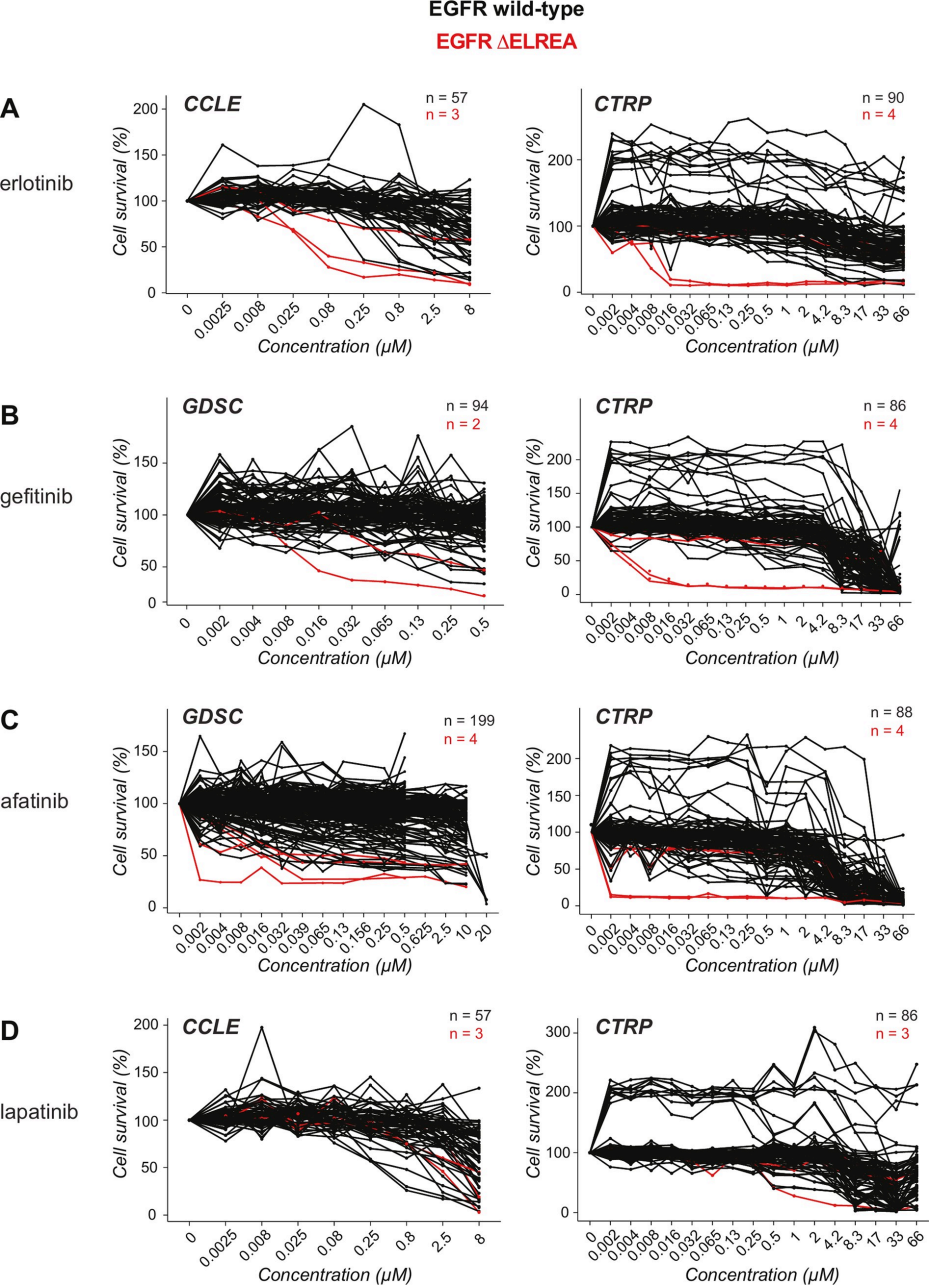
Response of EGFR ΔELREA mutant lung cancer cells to tyrosine kinase inhibitors. Sensitivity (cell survival versus inhibitor concentration) of lung cancer cell lines harboring EGFR ΔELREA mutant (red curves) versus wild-type EGFR (black curves) to (A) erlotinib, (B) gefitinib, (C) afatinib, and (D) lapatinib. The data were collected from the publicly available databases CCLE, CTRP and GDSC, as indicated.

The MDS results for the ΔELREA mutation are also consistent with the effects of compounds (Fig 10A–10C) that inhibit EGFR mutant cell lines [49–51]: inhibitors recognizing the active conformation are more effective against the ΔELREA mutant in lung cancer cell lines as compared to cell lines expressing wild-type EGFR. For example, the mean AUC values from CTRP data for wild-type and ΔELREA mutant were 13.45 and 8.20 with erlotinib (P < 0.0001 –derived using unpaired two-sample t-test), 12.43 and 6.98 with gefitinib (P < 0.0001), and 10.39 and 5.29 with afatinib (P < 0.0001), respectively. In contrast to these three EGFR inhibitors, with lapatinib (Fig 8D), which selectively recognizes and binds to the inactive form of the receptor [60], the mean AUC for wild-type EGFR (mean AUC 12.86) is not significantly different (P = 0.0844) from that of the ΔELREA mutant (mean AUC 11.28). Thus, the results of our study are consistent with the observed effects of conformation-specific EGFR ligands in cancer cell lines and further supported by simulations focused on inhibitor complexes with the active conformation of ΔELREA EGFR, where the restricted αC helix movement resulted in a more compact binding site impacting the interactions and affinity for inhibitors [61].

In conclusion, this study suggests that the effects exerted by ΔELREA are two-fold: the deletion mutation in EGFR increases kinase activity (1) by *stabilizing* that active catalytic conformation of the tyrosine kinase, and (2) by promoting a *conformational shift* towards the active catalytic state. Such “hot spot” somatic mutations, like ΔELREA, offer possibilities to select ligands that may block conformational and stabilization effects and hence provide possible therapeutic tools aimed at controlling ErbB phosphorylation and signaling. Indeed, known EGFR inhibitors that preferentially recognize the active kinase conformation, as promoted by the ΔELREA deletion, exert a more dramatic effect–reducing cell survival in cancer cell models–in comparison to conformationally malleable wild-type EGFR.

## Supporting information

S1 Table List of 3D structures used to build composite models for the MDS study. (PDF)

S2 Table Hydrogen bond interactions between wild-type/ΔELREA EGFR and ATP during MDS. (PDF)

S1 Fig Histogram plots of salt bridge distance, number of hydrogen bonds between EGFR and ATP, and free energy of binding of ATP from Figs 7B, 8B and 8C, respectively. (TIF)

S2 Fig Superposed coordinates of snapshots from each of the three ATP-bound replicate simulations of wild-type and mutant EGFR. (TIFF)

S1 File PDB coordinates of active form apo wild-type EGFR used for the simulation. (PDB)

S2 File PDB coordinates of active form apo ΔELREA EGFR used for the simulation. (PDB)

S3 File PDB coordinates of active form ATP bound wild-type EGFR used for the simulation. (PDB)

S4 File PDB coordinates of active form ATP bound ΔELREA EGFR used for the simulation. (PDB)

S5 File PDB coordinates of inactive form apo wild-type EGFR used for the simulation. (PDB)

S6 File PDB coordinates of inactive form apo ΔELREA EGFR used for the simulation. (PDB)

S7 File R code used to process the drug response data. (R)
