# Supplementary material for: Structural characterization of EGFR exon 19 deletion mutation using molecular dynamics simulation
Source: PLoS One. 2019 Sep 19;14(9):e0222814. doi: 10.1371/journal.pone.0222814 (PMC6752865; doi:10.1371/journal.pone.0222814)
Supplement: S1 Table — (PDF) [file pone.0222814.s001.pdf]

	Apo active EGFR	Apo inactive EGFR	ATP-bound active EGFR
**Principal structure**	2GS2 [1]	2GS7 [1]	2ITX [2]
**Structures used to build missing loops**	1M14 [3] (723-725)	3W2S [4] (958-984)	2GS6 [1] (862-865)
	3W2S [4] (967-981)	4HJO [5] (848-850)	3W2S [4] (990-1001)

- Numbers in bracket correspond to the range of amino acids missing from the principal structure which were then built using the indicated PDB structure.

References

1. Zhang X, Gureasko J, Shen K, Cole PA, Kuriyan J. An Allosteric Mechanism for Activation of the Kinase Domain of Epidermal Growth Factor Receptor. Cell. 2006;125(6):1137–49.
2. Yun C-H, Boggon TJ, Li Y, Woo MS, Greulich H, Meyerson M, et al. Structures of lung cancer-derived EGFR mutants and inhibitor complexes: mechanism of activation and insights into differential inhibitor sensitivity. Cancer Cell. 2007;11(3):217–27
3. Stamos J, Sliwkowski MX, Eigenbrot C. Structure of the epidermal growth factor receptor kinase domain alone and in complex with a 4-anilinoquinazoline inhibitor. J Biol Chem. 2002;277(48):46265–72.
4. Sogabe S, Kawakita Y, Igaki S, Iwata H, Miki H, Cary DR, et al. Structure-Based Approach for the Discovery of Pyrrolo[3,2- *d*]pyrimidine-Based EGFR T790M/L858R Mutant Inhibitors. ACS Med Chem Lett. 2013;4(2):201–5.
5. Park JH, Liu Y, Lemmon MA, Radhakrishnan R. Erlotinib binds both inactive and active conformations of the EGFR tyrosine kinase domain. Biochem J. 2012;448(3):417–23.
