# Supplementary material for: Structural characterization of EGFR exon 19 deletion mutation using molecular dynamics simulation
Source: PLoS One. 2019 Sep 19;14(9):e0222814. doi: 10.1371/journal.pone.0222814 (PMC6752865; doi:10.1371/journal.pone.0222814)
Supplement: S2 Table — (PDF) [file pone.0222814.s002.pdf]

(A) Hydrogen bond interactions between ATP and wild-type EGFR during the simulation

Acceptor	DonorH	Donor	Fraction	AvgDist (Å)	AvgAng
ATP@N1	MET_793@H	MET_793@N	0.9756	3.077	163.8957
GLN_791@O	ATP@HN61	ATP@N6	0.9668	2.904	157.554
ATP@O2B	LYS_745@HZ2	LYS_745@NZ	0.3312	2.8153	155.942
ATP@O2B	LYS_745@HZ3	LYS_745@NZ	0.312	2.8125	155.8344
ATP@O2B	LYS_745@HZ1	LYS_745@NZ	0.2664	2.8155	156.1433
THR_790@OG1	ATP@HN62	ATP@N6	0.1444	3.2063	144.6647
ATP@O2'	CYS_797@H	CYS_797@N	0.0736	3.0973	148.4985
ATP@O1B	ALA_722@H	ALA_722@N	0.0572	2.8777	159.0192
ATP@O3G	ARG_841@HH12	ARG_841@NH1	0.0128	2.8031	147.8435
ATP@N6	THR_790@HG1	THR_790@OG1	0.0112	3.3476	143.7813
ATP@O3G	ARG_841@HH11	ARG_841@NH1	0.0096	2.8841	158.3484
ATP@O3B	ALA_722@H	ALA_722@N	0.0072	3.3741	144.7664
ATP@O2G	LYS_875@HZ1	LYS_875@NZ	0.0064	2.8039	149.2692
ATP@O2G	LYS_745@HZ2	LYS_745@NZ	0.006	2.8975	148.5305
ASP_800@OD2	ATP@HO3'	ATP@O3'	0.0052	2.748	156.2707
ATP@O2G	LYS_875@HZ2	LYS_875@NZ	0.0048	2.7931	149.3414
ATP@O2G	LYS_875@HZ3	LYS_875@NZ	0.0036	2.7615	157.9891
ASP_800@OD2	ATP@HO2'	ATP@O2'	0.0028	2.7259	160.576
ATP@O2G	LYS_745@HZ1	LYS_745@NZ	0.0024	2.9571	149.3977
ATP@O1B	LYS_745@HZ2	LYS_745@NZ	0.0024	3.2319	138.4561
ATP@O3G	ARG_841@HH22	ARG_841@NH2	0.0016	2.8129	148.4546
ATP@O1B	LYS_745@HZ1	LYS_745@NZ	0.0012	3.1913	139.7704
ATP@O1B	LYS_745@HZ3	LYS_745@NZ	0.0008	3.082	137.5614
LEU_718@O	ATP@HO3'	ATP@O3'	0.0008	3.1931	149.6899
ATP@O3G	LYS_875@HZ3	LYS_875@NZ	0.0004	2.759	165.0465
ATP@O3G	LYS_875@HZ1	LYS_875@NZ	0.0004	3.1878	139.6111
ATP@O3B	LYS_745@HZ1	LYS_745@NZ	0.0004	3.2073	138.2237
ASP_800@OD1	ATP@HO3'	ATP@O3'	0.0004	3.3342	151.252
ATP@N6	MET_793@H	MET_793@N	0.0004	3.4578	146.4073
MET_793@N	ATP@HN61	ATP@N6	0.0004	3.4885	142.3253

(B) Hydrogen bond interactions between ATP and ΔELREA EGFR during the simulation

Acceptor	DonorH	Donor	Fraction	AvgDist (Å)	AvgAng
ATP@N1	MET_793@H	MET_793@N	0.9532	3.1257	160.9542
GLN_791@O	ATP@HN62	ATP@N6	0.898	2.9118	152.487
ATP@O3G	ARG_841@HH12	ARG_841@NH1	0.2856	2.8131	153.0624
ATP@O3G	ARG_841@HH22	ARG_841@NH2	0.2504	2.8237	151.3491
ATP@O2A	LYS_745@HZ2	LYS_745@NZ	0.196	2.8317	160.1432
ATP@O2B	LYS_745@HZ1	LYS_745@NZ	0.1696	2.775	157.0436
ATP@O2A	LYS_745@HZ1	LYS_745@NZ	0.1576	2.8509	160.5126
ATP@O2B	LYS_745@HZ2	LYS_745@NZ	0.1492	2.7799	157.8499
ATP@O2B	LYS_745@HZ3	LYS_745@NZ	0.1396	2.7816	155.9859
ATP@O2A	LYS_745@HZ3	LYS_745@NZ	0.1224	2.8502	159.5042
THR_790@OG1	ATP@HN61	ATP@N6	0.1192	3.1592	146.1309
ATP@O3G	ARG_841@HE	ARG_841@NE	0.1068	2.8846	158.6177
ATP@O2G	ARG_841@HH21	ARG_841@NH2	0.0948	2.8545	159.6861
ATP@O1B	ALA_727@H	ALA_727@N	0.0764	2.907	159.3931
ATP@O2'	CYS_797@H	CYS_797@N	0.0392	3.0809	149.0063
ATP@O3G	ARG_841@HH21	ARG_841@NH2	0.0388	2.9209	149.4318
ATP@O1B	LYS_745@HZ1	LYS_745@NZ	0.0376	3.0293	147.9211
ATP@O1B	LYS_745@HZ3	LYS_745@NZ	0.0364	2.9979	146.6659
ATP@O2G	LYS_875@HZ1	LYS_875@NZ	0.022	2.7695	157.0207
ATP@O1B	LYS_745@HZ2	LYS_745@NZ	0.0188	3.0996	146.4626
ATP@O3B	ALA_722@H	ALA_722@N	0.0172	3.3155	147.5082
ATP@O2G	ARG_841@HE	ARG_841@NE	0.0144	3.2149	140.7642
ATP@O2G	LYS_875@HZ3	LYS_875@NZ	0.0136	2.7549	158.395
ATP@O2G	LYS_875@HZ2	LYS_875@NZ	0.0132	2.8008	156.0448
ATP@O3G	LYS_875@HZ2	LYS_875@NZ	0.0112	2.8231	161.1563
ATP@O3G	LYS_875@HZ3	LYS_875@NZ	0.006	2.8272	158.5374
ATP@O2G	ARG_841@HH22	ARG_841@NH2	0.0052	2.966	161.2113
ATP@O3G	LYS_875@HZ1	LYS_875@NZ	0.0048	2.7511	156.6571
ATP@O1B	GLY_721@H	GLY_721@N	0.004	3.1913	142.8719
MET_793@N	ATP@HN62	ATP@N6	0.0028	3.3922	140.3938
ATP@N6	THR_790@HG1	THR_790@OG1	0.0024	3.2136	143.7323
ATP@O1A	LYS_745@HZ3	LYS_745@NZ	0.0008	2.8898	145.5669
LEU_718@O	ATP@HO3'	ATP@O3'	0.0008	3.1155	143.8834
ATP@O3A	LYS_745@HZ2	LYS_754@NZ	0.0008	3.4091	141.9069
ATP@O3A	LYS_745@HZ1	LYS_745@NZ	0.0008	3.4405	142.7711
ATP@N6	MET_793@H	MET_793@N	0.0008	3.4736	149.4192
ASP_800@OD1	ATP@HO3'	ATP@O3'	0.0004	2.6742	159.1776
ATP@O5'	LYS_745@HZ1	LYS_745@NZ	0.0004	3.0766	141.6863

ATP@O1A	LYS_745@HZ2	LYS_745@NZ	0.0004	3.1242	149.4501
ATP@O1B	PHE_723@H	PHE_723@N	0.0004	3.1336	156.1012
