# Supplementary figures and images for: Structural characterization of EGFR exon 19 deletion mutation using molecular dynamics simulation
Source: PLoS One. 2019 Sep 19;14(9):e0222814. doi: 10.1371/journal.pone.0222814 (PMC6752865; doi:10.1371/journal.pone.0222814)

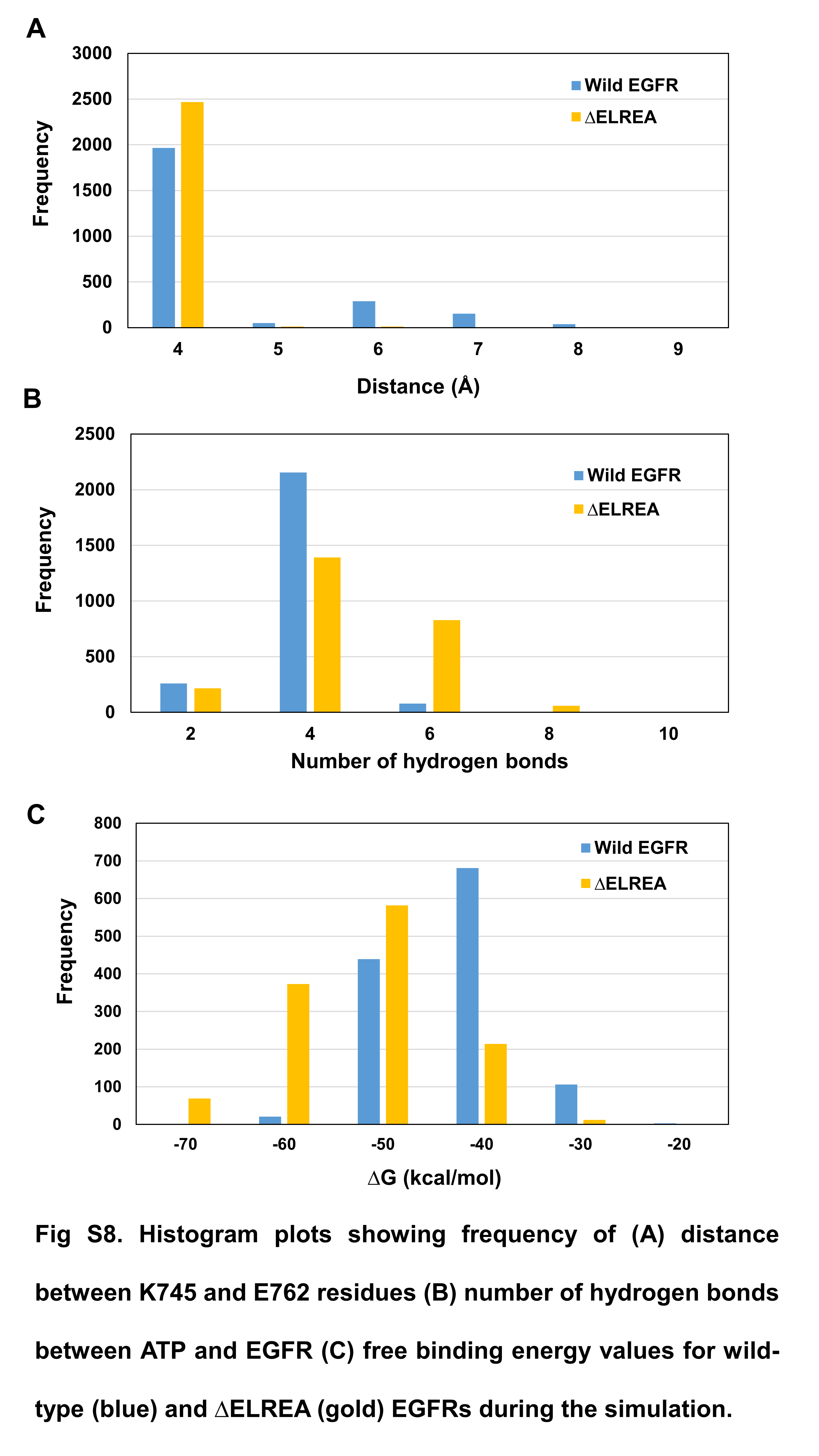

Supplement: S1 Fig — (TIF) [file pone.0222814.s003.tif]

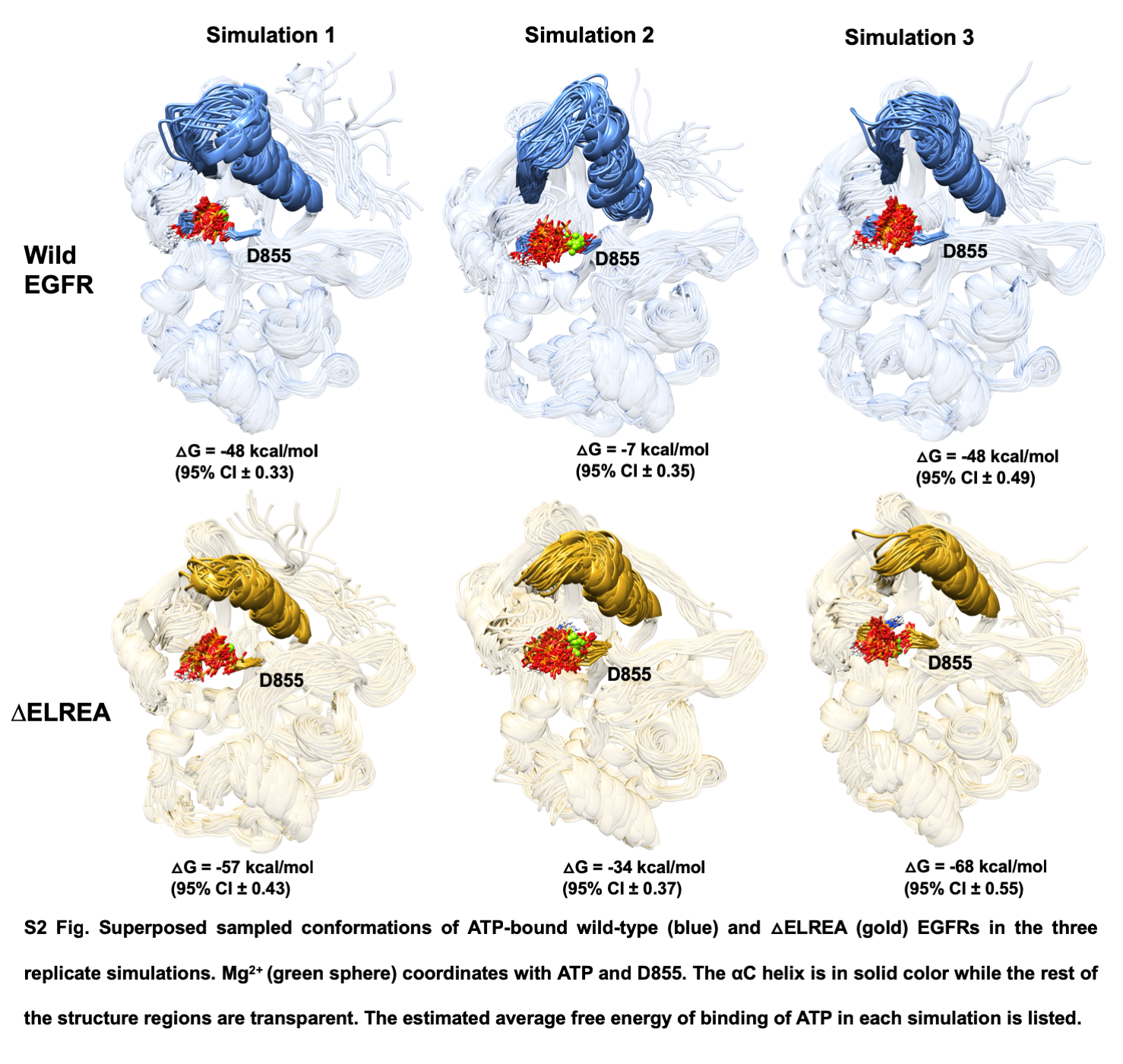

Supplement: S2 Fig — (TIFF) [file pone.0222814.s004.tiff]
